# Foraging-Based Enrichment Promotes More Varied Behaviour in Captive Australian Fur Seals (*Arctocephalus pusillus doriferus*)

**DOI:** 10.1371/journal.pone.0124615

**Published:** 2015-05-06

**Authors:** David P. Hocking, Marcia Salverson, Alistair R. Evans

**Affiliations:** 1 School of Biological Sciences, Monash University, Melbourne, Victoria, Australia; 2 Geosciences, Museum Victoria, Melbourne, Victoria, Australia; 3 Wild Sea Precinct, Zoos Victoria, Melbourne, Victoria, Australia; New York Institute of Technology College of Osteopathic Medicine, UNITED STATES

## Abstract

During wild foraging, Australian fur seals (*Arctocephalus pusillus doriferus*) encounter many different types of prey in a wide range of scenarios, yet in captive environments they are typically provided with a narrower range of opportunities to display their full repertoire of behaviours. This study aimed to quantitatively explore the effect of foraging-based enrichment on the behaviour and activity patterns displayed by two captive Australian fur seals at Melbourne Zoo, Australia. Food was presented as a scatter in open water, in a free-floating ball device, or in a static box device, with each treatment separated by control trials with no enrichment. Both subjects spent more time interacting with the ball and static box devices than the scatter feed. The total time spent pattern swimming was reduced in the enrichment treatments compared to the controls, while the time spent performing random swimming behaviours increased. There was also a significant increase in the total number of bouts of behaviour performed in all three enrichment treatments compared to controls. Each enrichment method also promoted a different suit of foraging behaviours. Hence, rather than choosing one method, the most effective way to increase the diversity of foraging behaviours, while also increasing variation in general activity patterns, is to provide seals with a wide range of foraging scenarios where food is encountered in different ways.

## Introduction

One of the challenges facing zoos and aquariums is the maintenance of species-typical and biologically-relevant behaviours performed by animals living in captivity [1]. Environmental enrichment is routinely used as a management tool to combat display of abnormal repetitive behaviours (e.g. repetitive pattern swimming in marine mammals) and in many cases has proved effective in reducing stereotypies, while also promoting increased activity levels and display of varied behaviours [2–6]. However, in many cases the methods used (e.g. provision of novel objects or toys) have little functional relevance to the animals and do not promote biologically-relevant behaviours similar to those used in the wild [7]. This is especially true in the case of marine mammals, with Hunter et al. [3] suggesting that “most marine mammal enrichment has focused on training, decreasing stereotypies, or providing behavioral changes to combat boredom, as opposed to promoting more natural behavior”. This stems from difficulties in identifying important natural behaviours, largely due to the challenges involved in making direct observations of wild animals at sea. Foraging behaviours are especially important because they represent some of the more varied and complex behaviours carried out in the wild, while also being intrinsically motivating and engaging for captive animals [8].

In this study we tested whether we could use foraging-based enrichment to increase the behavioural diversity displayed by two captive Australian fur seals (*Arctocephalus pusillus doriferus*). In the wild, Australian fur seals mostly perform benthic foraging, where they dive down and hunt for prey amongst the complex topography of the seafloor; however, they can also perform epipelagic foraging, where fast and evasive prey is pursued in open water [9–12]. We tested three methods of enrichment where the seals encountered the prey either free-floating in the water or concealed within a mobile or static enrichment device. Encountering free-floating prey was as close as possible to epipelagic foraging in open water, while the two forms of enrichment device were used to promote behaviours more similar to those that might be displayed when hunting cryptic prey among rocks or weeds during benthic foraging. From these results we conducted a detailed analysis looking at variation in the biomechanics and timing of the specific foraging behaviours displayed when capturing prey encountered using these three methods of prey presentation [13]. However, as well as promoting a wide range of prey capture and handling tactics, these three forms of enrichment also had an important effect on the general activity patterns and behaviours displayed by these seals. This is important when considering their potential for use as environmental enrichment tools for promoting more varied species-typical behaviour in captive seals and it is these results that we will report here.

## Methods

### Study Animals

To explore the behaviours displayed by captive fur seals we made detailed observations during feeding trials performed in the main seal pool in the ‘Wild Sea’ precinct of Melbourne Zoo (Elliott Ave, Parkville 3052, VIC, Australia). Two adult female Australian fur seals (*Arctocephalus pusillus doriferus*) were used during this study: Tarwin (ARKS# 980419 DOB: Est. November 1997) and Bay (ARKS# A70598 DOB: Est. November 2006). They lived on display as part of the permanent collection at Melbourne Zoo, where they had also been trained using operant conditioning to take part in educational displays for the zoo’s visiting public. All observations and protocols carried out as part of this work were done under the approval of the Monash University (FW/BSCI/2012/01) and Zoos Victoria (ZV12007) Animal Care and Ethics Committees.

### Data Collection

During the feeding trials one seal was given access to the main pool and left for 10 minutes to acclimate before the experimental session began. After acclimation, one of the seal’s keepers entered the exhibit and presented it with one of three enrichment treatments. We then filmed its behaviour from above and below the water for a 20-minute observation period. After the first seal completed its experimental session the animals were rotated so that the second individual could participate. The order of which seal participated first was haphazard and depended on which yard or pool each seal was in before the trials commenced that day. Each seal participated in only one experimental session per day and all sessions were carried out between 0730–1030 h before their first training session. Outside these trials the seals receive most of their daily diet in the form of fish presented as food rewards, which are hand fed to them during three training sessions spaced throughout the day. The seals are not fed overnight and so these experiments were the first foraging opportunity presented to the seal each day. It was hoped that this would help to ensure that they were hungry and motivated to engage with the different types of enrichment.

**Fig 1:**
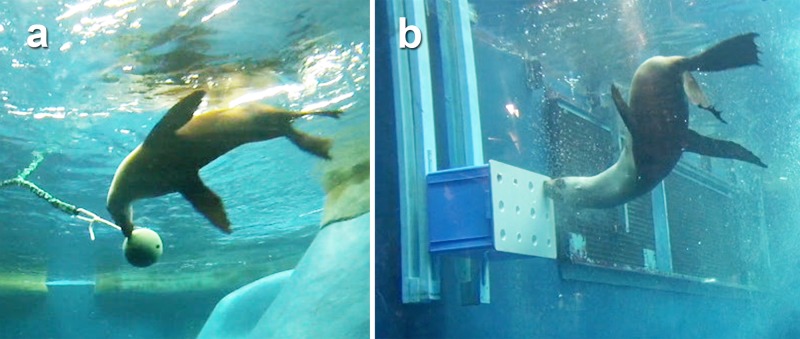
Mobile and Static enrichment devices. a) Mobile ball and rope enrichment device: fish are placed into the ball, which must then be manipulated by the seal in order to knock out the hidden prey items. b) Static box enrichment device: fish are hidden in the recessed tubes on the front surface of the device. Because the box device is static against the wall it cannot be manipulated to knock out prey items. See S1 Video for footage of the main behaviours used by the seals when interacting with these devices.

In each enrichment treatment session we presented the seal with six dead fish (three whiting *Sillago* sp. and three pilchards *Sardinops* sp.), totaling approximately 200–300 grams. Adult female seals have a daily food intake (DFI) of between 3.5–4 kg. The number of fish used in these trials therefore constituted only a small fraction of their daily diet (approximately 5–8%). We chose to use six small fish of the same two species so that any variation in the seal’s behaviour could be related to change in the presentation method using the three forms of enrichment, rather to differences in the prey species presented. The first enrichment treatment involved throwing a scatter of prey to random locations around the pool so that the seal encountered them free-floating in the water (scatter feed treatment). The second enrichment treatment involved presenting the six prey items inside a ball enrichment device attached to an elastic bungee cord (mobile ball device treatment; Fig 1a). This required the seal to manipulate the object to knock out the prey hidden within it. Both scatter feeds and the mobile ball enrichment device are used regularly at Melbourne Zoo as forms of foraging based enrichment; however, until now their effectiveness has not been quantitatively assessed. In contrast, the third enrichment method used a new type of device developed for this study, where the seal was presented with prey concealed within a static box enrichment device (static box device treatment; Fig 1b). This device was developed based on an experimental apparatus used by Hocking et al. [14] to test the suction feeding abilities of captive leopard seals (*Hydrurga leptonyx*). The device was made from a plastic storage box with a plastic front-plate into which we set recessed PVC tubes. The box was fixed to a wooden frame that was tied to the pool fence using Velcro straps. Fish were placed into the recessed tubes and a plastic board was positioned over the front of the device to hold in the fish as the device was lowered into the water. Unlike the ball device, the static box could not be manipulated to knock out the prey items because it was securely fixed to the fence. We also performed control experimental sessions, where the seal was released into the pool, but no enrichment was presented during the 20-minute observation period. This allowed us to compare the behaviour patterns displayed both in the presence and absence of foraging-based enrichment.

These four treatment types (scatter feed, mobile ball device, static box device and control with no enrichment) were each presented to the seals five times in a row on separate days to form a group or block of five replicates for each method (Table 1). As part of the experimental design we carried out a block of five control sessions before and after each block of five enrichment treatment sessions. This allowed us to ensure that the there was no flow-on effect from one enrichment-treatment block to the next. As a result, each seal was presented with a repeated measures design involving three enrichment treatment blocks separated by four control blocks: Control A, Enrichment 1, Control B, Enrichment 2, Control C, Enrichment 3, Control D (Table 1). These trials were scheduled to be performed twice a week commencing in September 2012. Weather events and unforeseen activities at the zoo meant that in some weeks it was not possible to carryout trials on the scheduled days, extending the duration of the trials, which were completed by March 2013.

**Table 1:** Summary of the experimental design used and total time of observation.

*Treatment*	*Procedure*	*Replicates*	*Total time of observation*
Control A	No enrichment presented	5 x 20 min	100 min
Enrichment 1	Scatter feed treatment	5 x 20 min	100 min
Control B	No enrichment presented	5 x 20 min	100 min
Enrichment 2	Mobile ball device treatment	5 x 20 min	100 min
Control C	No enrichment presented	5 x 20 min	100 min
Enrichment 3	Static box device treatment	5 x 20 min	100 min
Control D	No enrichment presented	5 x 20 min	100 min

The 20-minute observation session for both control and treatment sessions was recorded using five high-definition video cameras (one Sony NX70, four GoPro HERO2) placed around the enclosure to capture a near-complete visual record of the seal’s behaviour. Two cameras were placed above the water, while the remaining cameras viewed the pool through underwater viewing windows. The footage was compiled into a single video frame using Adobe Premiere Pro CC v7.1.0 (Adobe Systems Inc., San Jose, California) and exported as a QuickTime movie file (.MOV) for further analysis. After both seals had completed their experimental sessions for the day they re-commenced their normal daily routine, starting with a training session where they received the remainder of their morning food allowance.

### Statistical Analysis

We created an ethogram based on the behaviours displayed during *ad libitum* observation sessions conducted before the trials commenced and on the behaviours displayed during the trials (Table 2). The video file for each session was viewed in Adobe Premiere Pro CC and text markers were placed onto the footage to mark the start and end of each bout of behaviour. The video time-code was then used to calculate the duration of each bout, allowing us to calculate the total time spent performing each type of behaviour for every session. These data were used to compare how much time was spent interacting with the three enrichment treatments, as well as allowing us to compare the amount of time spent performing repetitive pattern versus random swimming behaviours during the treatment and control sessions (see operational definitions in Table 2). Unequal variance in the times spent performing these behaviours led us to use non-parametric Kruskal-Wallis tests followed by Mann-Whitney U post hoc tests to look at pairwise comparisons against a critical value of α = 0.05. A Kruskal-Wallis test was also performed on the total number of bouts of behaviour performed by the seal during each session. All statistical analyses were conducted using R statistical and graphical environment (R version 3.0.2, R development core team, 2013) [15].

**Table 2:** Ethogram and operational definitions for Australian fur seal behaviour during the experimental sessions carried out in this study.

*Behaviour category*	*Grouped behaviours*	*Description*
Foraging enrichment	Enrichment	Exploring and interacting with enrichment device in search of prey items. Includes looking at, blowing bubbles and touching the device with muzzle/whiskers, as well as handling of prey items if not swallowed immediately upon capture.
Non-foraging enrichment	Enrichment	Exploration or interaction with the enrichment device in parts where no food was present.
Pattern swimming	Pattern Swimming	Smooth swimming style at a near constant speed in a repetitive pattern for two or more complete revolutions of the pattern to complete “laps” of a part of the pool.
Random swimming	Random Swimming	All other non-repetitive swimming around pool including general locomotion such as swimming between bouts of enrichment use or grooming.
Fast swimming	Random Swimming	Swimming at a fast speed around the pool. This was generally performed when searching the pool for prey during enrichment treatments (especially during the scatter feed) or to gain speed prior to hauling out of the water.
Head out of water	Random Swimming	A sub-category of random swimming where the seal is swimming or floating at the surface with head clear of the water while watching or looking for something above the surface.
Exploration	Other	Investigating or exploring the gates, sides and bottom of the pool or floating non-enrichment objects (such as tree leaves or feathers) by closely looking at them or touching them with their muzzle or whiskers.
Grooming	Other	Maintenance behaviours performed while floating at the water’s surface including scratching and wiping face or body with flippers.
Hauled out	Other	Hauled out of water onto rocks around pool.
Interaction with observer	Other	Watching or interacting with the observer, seal keepers or other zoo staff as they moved around the pool.
Out of sight	Other	Seal was out of sight behind the island in the center of the pool (the only area not visible from any of the camera views).

Behaviours recorded include all major behavioural states observed during these trials, but do not constitute a complete list of behaviours displayed by Australian fur seals in captivity. See S1 Video for footage of some of these behaviour states.

## Results

### Foraging Behaviours and Time Spent Interacting with Enrichment

The foraging behaviours used by Bay and Tarwin to capture prey differed between the three enrichment treatments. When capturing free-floating prey during the scatter feed both subjects used raptorial biting, where the prey item was captured in the teeth. But biting alone could not be used to capture prey concealed within the two enrichment devices. The mobile ball device was pushed with the muzzle until the fish fell out. This was assisted by using suction feeding to help draw out concealed prey items. When using the static box device the seals could not manipulate or push the box to knock out the prey items. Here, suction feeding was the main tactic used to draw out concealed prey. Tarwin also blew a stream of bubbles into the recessed tubes in the box device to knock out hidden prey. For further information on the biomechanics and timing of the specific foraging behaviours used in these trials see Hocking et al. [13].

**Fig 2:**
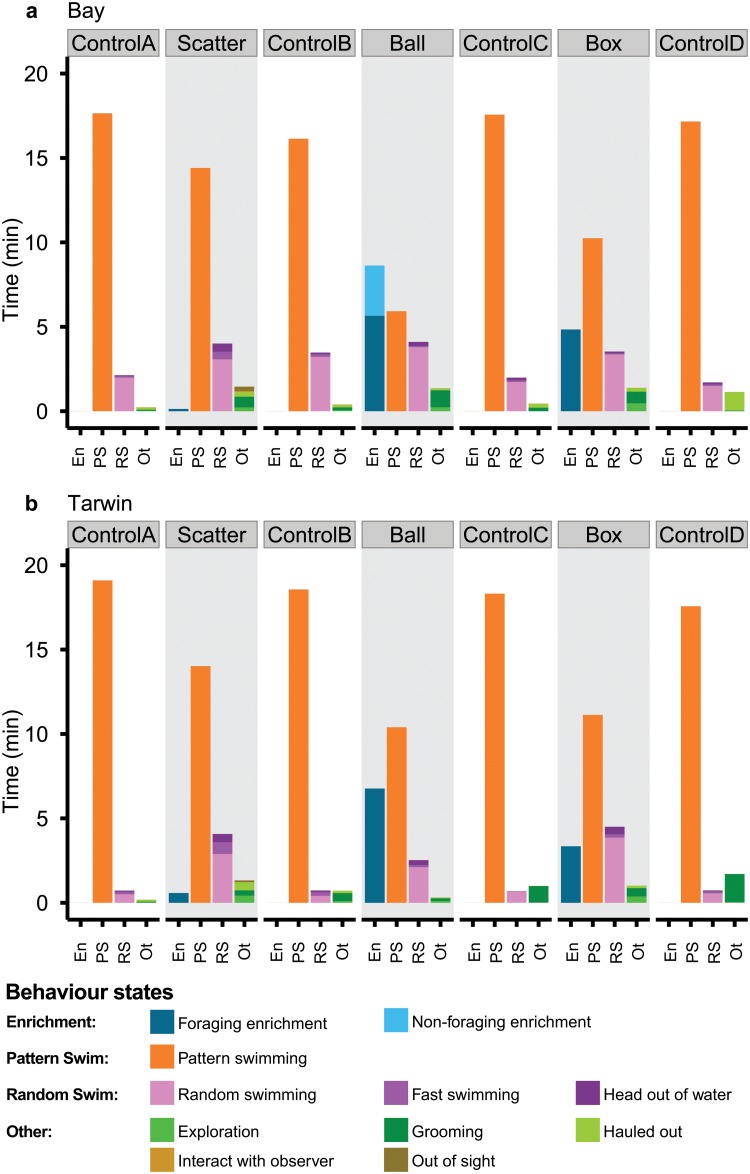
Summary of behaviours displayed during feeding trials. Mean duration (min) of behaviour states performed by Bay (a) and Tarwin (b) during each control and enrichment treatment. Similar behaviour states are grouped to show time spent performing: enrichment-related behaviours, pattern swimming, random swimming or any other behaviour state. Operational definitions for behaviour states are outlined in Table 2.

The total time spent interacting with the three enrichment treatments was also significantly different for both Bay (χ^2^ (2) = 10.82, p = <0.01) and Tarwin (χ^2^ (2) = 7.5, p = 0.02; Fig 2). Bay spent the most time interacting with the ball enrichment device, spending on average 8.6 ± 1.7 minutes (mean ± se; S1 Fig). This was not significantly different to the time spent using the static box enrichment device, where she spent an average of 4.84 ± 1.3 minutes interacting with the enrichment. Significantly less time was spent interacting with the scatter feed (0.14 ± 0.03 minutes). Here, the fish could be very quickly captured and consumed because they were not concealed within a device. When interacting with the ball device Bay showed two distinct types of behaviour. When searching for prey she actively manipulated and pushed the plastic ball part of the device, while looking into the hole in its side to locate the fish. However, Bay also spent 29 ± 7.55% (mean ± se) of her time interacting with the ball device by holding it by the rope and shaking it at or near the water’s surface as a form of object-play (Fig 2; S1 Video).

Tarwin also spent more time interacting with the ball device (6.7 ± 1.97 minutes) and static box enrichment device (3.35 ± 0.68 minutes) compared to the scatter feed (0.58 ± 0.3 minutes). Time spent using the static box device was significantly different to that spent interacting with the scatter feed; however, it was not significantly different to the time spent using the mobile ball device (S1 Fig). Time using the ball device was not significantly different to the scatter feed. This is likely due to one ball session in which Tarwin spent a short time interacting with the ball before ignoring it for the rest of the session without capturing any of the hidden fish. Unlike Bay, Tarwin interacted with the ball device only while searching for prey and did not shake the ball at the surface.

### Total Time Spent Pattern Swimming

The total time spent performing pattern swimming was not significantly different between the four control blocks for either individual (Bay: χ^2^ (3) = 2.75, p = 0.43, Tarwin: χ^2^ (3) = 2.75, p = 0.43) and so these were combined into a single treatment for comparison with the enrichment treatments. For both individuals the total time spent performing pattern swimming was significantly affected by the use of enrichment (Bay: χ^2^ (3) = 23.15, p = <0.01, Tarwin: χ^2^ (3) = 21.57, p = <0.01). In both cases the total time spent pattern swimming was significantly less in the enrichment treatments compared to the controls, but was not significantly different between the three forms of enrichment (Fig 2). Bay performed pattern swimming for on average 14.41 ± 0.78 (mean ± se) minutes during the scatter feed, 5.9 ± 1.8 minutes during the ball treatment and 10.24 ± 1.31 minutes during the static box treatment (S2 Fig). In contrast she performed significantly more pattern swimming in the control sessions (17.12 ± 0.9 minutes). Tarwin performed pattern swimming for on average 14.02 ± 1.19 minutes during the scatter feed, 10.39 ± 2.1 minutes during the ball treatment and 11.13 ± 1.11 minutes during the static box treatment (S2 Fig). In contrast, during the controls, Tarwin performed significantly more pattern swimming (18.38 ± 0.46 minutes).

### Total Time Spent Performing Random Swimming

Use of enrichment also affected the total time spent performing random swimming behaviours. Total time spent performing random swimming was not significantly different between the four control blocks for either Bay (χ^2^ (3) = 3.86, p = 0.28) or Tarwin (χ^2^ (3) = 0.3, p = 0.96). These were grouped together for comparison with the enrichment treatments for each individual.

When comparing the three enrichment treatments and the controls, the treatments did have a significant effect on the total time spent performing random swimming for both Bay (χ^2^ (3) = 12.48, p = 0.01) and Tarwin (χ^2^ (3) = 21.56, p = <0.01; Fig 2). Bay performed random swimming for on average 4.0 ± 0.71 (mean ± se) minutes during the scatter feed, 4.1 ± 0.92 minutes during the ball treatment and 3.53 ± 0.25 minutes during the static box treatment. In contrast she performed random swimming for only 2.32 ± 0.62 minutes during the controls. However, only the static box treatment and controls were significantly different from one another (S3 Fig).

Tarwin performed random swimming for on average 4.08 ± 0.98 minutes during the scatter feed, 2.53 ± 0.51 minutes during the ball treatment and 4.51 ± 0.48 minutes during the static box treatment. During the controls she performed random swimming for on average 0.72 ± 0.31 minutes. A significant difference was found between the controls and both the ball and static box enrichment treatments. However, the scatter feed treatment was not significantly different than the controls (S3 Fig).

### Number of Bouts of Behaviour

As well as differing in the total time spent performing pattern and random swimming, both seals also differed in the number of bouts of behaviour performed during control and enrichment treatments. This indicates how often the seals changed behaviours during the observation period. Firstly, the four control blocks were not significantly different from each other for either Bay (χ^2^ (3) = 2.67, p = 0.45) or Tarwin (χ^2^ (3) = 2.55, p = 0.47) and so were grouped into a single treatment for comparison with the enrichment treatments. For both individuals the treatment had a significant effect on the number of bouts of behaviour performed (Bay: χ^2^ (3) = 23.56, p = <0.01, Tarwin: χ^2^ (3) = 23.94, p = <0.01). Post hoc tests revealed that for both seals, significantly more bouts of behaviour were performed during the enrichment treatments than during the controls (S4 Fig). There was no significant difference in the number of bouts performed during the three enrichment treatments. When we group the three enrichment treatments together we find that Bay performed on average 46 ± 3.35 (mean ± se) bouts of behaviour during the enrichment treatments, but only 18 ± 1.59 bouts of behaviour during the controls. Tarwin performed 39.07 ± 3.88 bouts of behaviour during the enrichment treatments and only 11.5 ± 1.32 bouts during the control sessions. Hence, both seals alternated between behaviours significantly more in the presence of enrichment than in the controls where no enrichment treatment was presented.

## Discussion

These results indicate that foraging-based enrichment can be effectively used to increase the variation in both the specific foraging tactics used and in the general activity patterns displayed by the seals. The mobile ball and static box devices resulted in more time spent interacting with the enrichment compared to the scatter feed treatment. This makes sense as the prey items could be quickly and easily captured and consumed when found in open water during the scatter feed, while more time had to be spent manipulating the ball device and searching the static box before the concealed prey could be retrieved. These three methods of enrichment also promoted a diverse range of foraging behaviours that likely reflect some of the tactics employed when targeting prey in the wild [13]. During the scatter feed, prey was captured using biting, which is likely similar to how epipelagic prey items are captured in the wild. When using the two enrichment devices the seals were required to actively search the devices before using a combination of suction feeding and biting to draw out and capture the prey items. While the ball device could be manipulated to knock out the concealed prey, the static box device could not. In this way the static box device likely provided the seal with opportunity to display behaviours similar to those used when hunting cryptic prey over a rocky seafloor in the wild. Hence, use of these three forms of enrichment each provided the seals with behavioural opportunities that allowed them to display different components of their wild feeding repertoire.

Increase in time spent interacting with enrichment treatments led to a significant decrease in time spent pattern swimming compared to the controls, which supports previous studies on pinnipeds that found that environmental enrichment can be effectively used to reduce the frequency and severity of repetitive stereotypic behaviours [2–6]. However, it should be noted that the performance of pattern swimming in these seals is not necessarily a sign of negative wellbeing. Newberry [7] notes that, “Some behaviour currently considered abnormal may in fact be adaptive in captivity, conferring a selective advantage on individuals performing the behaviour”. In the wild Australian fur seals spend long periods of time swimming in the open ocean when traveling between haul out and foraging sites [11]. Hence, long periods of time spent continuously swimming in captivity should not necessarily be considered abnormal. Franks et al. [16] suggested that individual swimming patterns in captive walrus (*Odobenus rosmarus*) might have a functional role related to the amount of time spent swimming in the wild and the formation of “microterritories” within the captive environment. These microterritories could enable the animals to swim at a preferred swimming frequency while also minimizing any potential collisions or antagonistic interactions with other animals in the pool. We could not assess this with our data as both seals were tested individually; however, it is interesting to note that both Bay and Tarwin displayed different swimming patterns. It would be very interesting to look more closely at the shape and timing of swimming patterns between animals within a stable social group, to see if it provides insights as to whether these patterns help to maintain separation between individuals.

As well as reducing pattern swimming, this study also found that the time spent performing random swimming behaviours was significantly higher in the static box enrichment treatments (and ball treatment for Tarwin) when compared to the control sessions. The seals were more actively engaged with their environment during random swimming, which occurred when moving between areas of interest (e.g. haul out sites, gate and enrichment devices), than they were when performing a repeating swimming pattern where they showed no signs of active engagement or interest in any part of their environment.

Both seals also performed a significantly higher number of bouts of behaviour in the presence of enrichment compared to the controls. During control sessions the seals often commenced pattern swimming near the beginning of the session and performed this behaviour for long stretches without changing behaviour. In contrast, during the enrichment treatments the seals frequently alternated between behaviour states, even after they had captured all of the prey items provided. For example, when presented with the static box device the seals frequently came back to explore or check the device for more prey, even after they had captured all available prey items. The seals also frequently explored the pool floor and walls following their use of the enrichment, seemingly in search of missed prey items (S1 Video). This included actively investigating any floating objects found, such as tree leaves or feathers that had fallen into the pool. In contrast, during the control sessions the seals showed little interest in investigating their environment, even when the observers had noticed some items floating in the pool. It is important to note that the number of bouts was not found to be significantly different between the three enrichment treatments, even though the duration of direct interaction differed dramatically between the three treatments. Hence, even a short duration of direct interaction with enrichment (as seen in the scatter feed treatments) can significantly affect their longer-term activity patterns in a positive way by making them more engaged with their environment. However, given the short duration of the feeding trials in these experiments further research should look at whether these effects are maintained over longer time periods.

Use of enrichment likely has welfare benefits for the animals by providing them with positive behavioural opportunities. The static feeding box device in particular presents the seals with a problem-solving task where they must explore the device and use trial and error to learn how best to find and draw out hidden prey. By varying the location of the prey items across the 12 hiding places between treatments, we can maintain a certain level of complexity even after they have learned how to efficiently extract prey from the device. This type of problem-solving task has been associated with positive affective states that can be used as indicators of positive animal wellbeing [17]. An extension of this project could involve creating a more complex static enrichment device with more hiding places positioned over a naturalistic surface (e.g. an artificial rock pile with crevices and seaweed). As well as providing enrichment to the seals, this could also be used to study their cognitive function as recommended by Clark et al. [18] by allowing us to look at how they go about searching a complex foraging patch. In our study both subjects seemed occasionally to search semi-systematically by moving across the static box device looking down each tube in turn. The ability to remember where they have previously searched would be invaluable during benthic feeding in the wild when searching for prey over large areas and across multiple dives. It would also be interesting to see if more complex foraging behaviours could be promoted by allowing the seals to interact with different types of seafloor substrates concealing hidden prey. Kastelein and Wiepkema [4] provided walruses with different substrate types in a stainless steel digging trough to encourage rooting behaviours. The extent to which Australian fur seals root or dig in seafloor sediments for prey is unknown, so it is unclear whether this type of enrichment would be worthwhile for captive Australian fur seals. Their whiskers do not wear down heavily from contact with the seafloor like wild walruses, indicating that this behaviour is not commonly performed; however, wild harbour seals (*Phoca vitulina*) have been observed digging in sediment for sand lance (*Ammodytes dubius*) using their flippers and Hawaiian monk seals (*Monachus schauinslandi*) are known to overturn rock fragments in search of cryptic prey like eels [19–21].

Interestingly, as well as promoting a more diverse range of foraging behaviours, use of the mobile ball enrichment device also provided Bay with the opportunity to perform object play, where the device itself was shaken at the water’s surface (S1 Video). This behaviour appears to be very similar to object play performed in the wild, where young Australian fur seals are commonly seen to thrash seaweed at the surface while playing in rock pools around seal colonies (S1 Video). This thrashing behaviour is also similar to the prey processing method used by Australian fur seals when processing prey that is too large to swallow whole, so it is possible that young seals perform this type of object play as a way of practicing foraging tactics they will use later in life.

This study helps to illustrate how vital it is that we assess the relative effectiveness of different forms of enrichment in an empirical manner. It is only by directly measuring the detailed behavioural responses that we can make accurate comparisons between the outcomes of different methods [22]. By making direct measurements of the animal’s behaviour when using the three forms of enrichment in this study, we have been able to quantitatively compare the specific behaviours displayed, the duration of direct interaction and affect of the enrichment on their general activity patterns. This not only helps us to assess the effectiveness of these specific methods, but also provides a measured baseline of behaviour that we can look at when assessing the enrichment value of other stimuli that the animals encounter during their daily routine. As well as using enrichment devices, the seals are also provided with a wide range of prey species that differ in size and shape, which can also promote a more diverse range of foraging behaviours. Most of this food is provided as rewards hand-fed to them during training to take part in educational displays and so that they can participate willingly in their husbandry and veterinary care. This training can also be considered a type of enrichment [23]. They also have less formal enrichment in the form of environmental stimulation and direct interaction with keepers and divers that enter the pool to maintain and clean the facility. Outside these trials the seals are also usually in the pool together, increasing their conspecific social interactions. This study provides us with a better understanding of what to look for when informally assessing the animal’s behaviour during their routine care, and also provides a starting point for further research into the specific effects of these other sources of environmental stimuli.

Empirical measurement of behaviour also provides an important baseline that allows us to look at the effectiveness of enrichment methods through time for the same individuals. This is especially important in the case of the static box device, which was developed for this study and hence can be considered novel to these animals. By carrying out a similar observation procedure in the future, we can quantitatively compare whether the animal’s engagement with this device changes as it becomes less novel. In contrast it is not possible to assess whether the seal’s engagement with the mobile ball device has changed through time, as their first interactions with this device were not recorded.

One of the potential problems with using foraging-based enrichment is that it uses up part of their daily food intake (DFI). To keep the seals healthy their diet is strictly controlled so that they get enough nutrients while not becoming overweight. An adult female seal like Tarwin has a daily food allowance of between 3.5 and 4 kg of fish. Because each animal only has a finite amount of food available per day, any foraging-based enrichment presented uses up part of their DFI. This can become an issue because food rewards are an integral part of their daily training routine, including being necessary for moving animals between pools and holding yards. This limits the amount of food available to be presented as part of enrichment activities outside of their training. It is therefore important to use enrichment methods that maximise the duration of direct engagement while using as little food as possible. For example the scatter feed used in this trial used the same amount of fish as the other two methods, but had a much shorter duration of direct interaction with the enrichment. In all three methods we only used six fish, totaling approximately 200–300 g. This means that for Tarwin we were only using between 5–8% of her DFI. One way to improve this further would be cutting fish into smaller pieces. This would give the seals opportunity to perform more prey captures and spend more time searching for more prey, while getting the same amount of food. However, this would likely also have other consequences. Smaller prey items in the mobile ball device might fall out much quicker as it is manipulated compared to whole fish, shortening the duration of engagement with the enrichment before all prey items are captured. In contrast though, more individual prey items placed into each tube of the static box device would likely increase the amount of time spent searching the device. This could also be improved upon further through addition of artificial seaweed that makes it more difficult to visually locate the prey items in the device. However, if the prey is too small then the seals may not consider it worth the search effort.

This study showed that by varying how we present food to captive fur seals, we can promote a more diverse range of foraging behaviours that are likely similar to some of those displayed when hunting in the wild. These methods of foraging-based enrichment can therefore be considered useful as tools for promoting a greater range of biologically relevant and species typical behaviours in captivity. These methods also had an important effect on the seals’ general activity patterns, increasing the number of bouts of behaviour while increasing the performance of random swimming and decreasing the performance of repetitive swimming patterns. These are positive outcomes that have important implications for the welfare and well-being of these animals. By quantitatively testing and documenting the effectiveness of these methods of enrichment, we hope that these results can be useful for others when designing enrichment protocols intended to encourage a greater range of biologically relevant behaviours in captive pinnipeds.

## Supporting Information

S1 Fig Total time spent interacting with the three enrichment treatments. Total time spent interacting with the scatter feed treatment, mobile ball and rope enrichment device and static box enrichment device for both Bay (a) and Tarwin (b). Boxplots with different letters are significantly different based on post hoc Mann-Whitney U tests with Bonferroni correction (α = 0.05).
(EPS)

S2 Fig Total time spent pattern swimming. Total time spent pattern swimming during each of the three enrichment treatments (scatter feed, mobile ball and rope enrichment device and static box enrichment device) and the control, for both Bay (a) and Tarwin (b). Boxplots with different letters are significantly different based on post hoc Mann-Whitney U tests with Bonferroni correction (α = 0.05).
(EPS)

S3 Fig Total time spent performing random swimming. Total time spent random swimming during each of the three enrichment treatments (scatter feed, mobile ball and rope enrichment device and static box enrichment device) and the control, for both Bay (a) and Tarwin (b). Boxplots with different letters are significantly different based on post hoc Mann-Whitney U tests with Bonferroni correction (α = 0.05).
(EPS)

S4 Fig Number of bouts of behaviour. Number of bouts of behaviour performed in each session compared between the controls and the three enrichment treatments. This indicates how many times the seals changed between behaviour states during each session. Boxplots with different letters are significantly different based on post hoc Mann-Whitney U tests with Bonferroni correction (α = 0.05).
(EPS)

S1 Video Behaviours performed by Australian fur seals (*Arctocephalus pusillus doriferus*) in response to each enrichment treatment. (MOV)
